# Interlayer rotation (twisting) switches interlayer mobility in moiré superlattices

**DOI:** 10.1073/pnas.2612453123

**Published:** 2026-09-22

**Authors:** Xuejiao Zhang, Jimin Ham, Vesna Srot, Peter A. van Aken, Kelvin Anggara

**Affiliations:** ^a^ Max Planck Institute for Solid State Research, Stuttgart 70569, Germany

**Keywords:** atomic-scale dynamics, structure–property relationship, surface lubricity

## Abstract

Mobility of mechanical contacts is widely attributed to atomic structures at the interface between contacting bodies. Matching (commensurate) interfacial structures are thought to reduce mobility, while mismatched (incommensurate) structures are thought to increase mobility. Despite its wide acceptance, direct observation of such structure–properties relationships remains elusive today due to limited microscopy access to atomic motions at buried interface between two bodies. Here, we capture key structural transitions linking interfacial mismatch and mobility in real-time by following twisting and sliding dynamics in bilayer moiré nanoflakes of MoS_2_ with atomically resolved transmission electron microscopy. The atomic motions show that slippery, mismatched interfaces—unlike sticky, matching interfaces—exhibit distinct dynamical signatures, such as tenfold increase in interfacial mobility and desynchronized atomic motions.

## Significance

When two contacting bodies move against one another, friction at interface dictates how much kinetic energy is dissipated as heat. A key assumption in understanding friction (tribology) is that atomic registry at the interface governs interfacial mobility: Atomically matched (commensurate) contacts resist motion, whereas atomically mismatched (incommensurate) contacts enable motion with minimal resistance. However, direct experimental validation of this relationship has remained elusive because the relevant atomic motions at buried solid–solid interfaces are inaccessible to microscopy. Here, using stacked nanoflakes of layered materials, we demonstrate how interfacial commensurability controls whether a contact is sticky or slippery at the atomic level. Beyond addressing a long-standing challenge in nanotribology, our findings open pathways to engineer superlubric contacts and dynamic moiré devices.

Solid–solid interfaces formed by contacting two solids exhibit ranges of unusual atomic geometries often inaccessible in bulks, which provide a rich platform to explore condensed matter phenomena (1–8). One such phenomenon is superlubricity or “structural lubricity,” where two surfaces with mismatched lattices slide against each other with negligible friction (1–3), offering a way to minimize wear and energy loss in mechanical contacts without lubricants. First predicted in 1983 (9, 10), superlubricity has since been observed in systems ranging from layered materials (11–19) to molecule–surface (20–22) and nanocrystal–surface (23, 24) interfaces, spanning length scales from nanometers to centimeters. While these works attribute the observed high interfacial mobility to atomic lattice mismatch at the contact interface, direct observation linking between interfacial atomic structures and mobility remains elusive (25). Establishing this link is challenging due to limited microscopy access to buried interfaces between two bulk solids and the simultaneous need to track interfacial mobility in real time (26, 27).

Moiré superlattices formed by stacking two-dimensional materials at specific relative orientations (i.e., twist angle, θ_twist_) provide an attractive platform to investigate links between interfacial structures and mobility at the atomic scale (28). Varying θ_twist_ in moiré superlattices, on top of accessing exotic electronic properties (5–7), also provides access to both matching (commensurate) and mismatched (incommensurate) contact interface between the layers, whose atomic structures can be followed in real-space and real-time using electron microscopy (7). However, common preparation of moiré superlattices by exfoliation or epitaxial growth often yields moiré domain a few microns in size (7) that tend to distort (29), ripple, and fold (30), thus creating a zoo of heterogeneous structures that obscure the link between interfacial structures and mobility. In addition, the large micron-scale moiré domain creates challenges for manipulating θ_twist_ as well as real-time tracking of individual atoms (e.g., ~22 million Mo-atoms for 1 μm^2^ of bilayer MoS_2_). Hence, the challenge of connecting interfacial structures and mobility requires means to prepare ripple-free moiré domains at the nano-scale, suitable for real-time atomic tracking and also for θ_twist_ manipulation.

Here, we present direct visualization of atomic dynamics linking interfacial structures to interfacial mobility in moiré superlattices, enabled by the preparation and direct imaging of nanoscale bilayer MoS_2_ moiré superlattices. By following motions of individual atoms in the moiré superlattice using transmission electron microscopy, we accessed the atomic-resolved dynamics pertinent to interfacial mobility, such as interlayer rotation (i.e., twisting) and interlayer translation (i.e., sliding). We visualized these interlayer dynamics across various commensurate and incommensurate contact geometries of the moiré superlattice, accessed via electron-induced manipulation of θ_twist_. Our observations, supported by Density Functional Theory (DFT) calculations, reveal signatures of high interlayer mobility (slippery) states, seen exclusively in incommensurate interlayer contacts, such as enhanced sliding and interlayer depinning. Also, our work reveals the signatures of low interlayer mobility (sticky) states, seen exclusively in commensurate contacts, such as restricted sliding and interlayer pinning. These atomic-scale dynamics unequivocally establish the key role of interfacial structures in switching interlayer mobility on and off, which opens additional strategies in engineering macroscopic superlubric surfaces and dynamic twistronics devices.

**Fig. 1.**
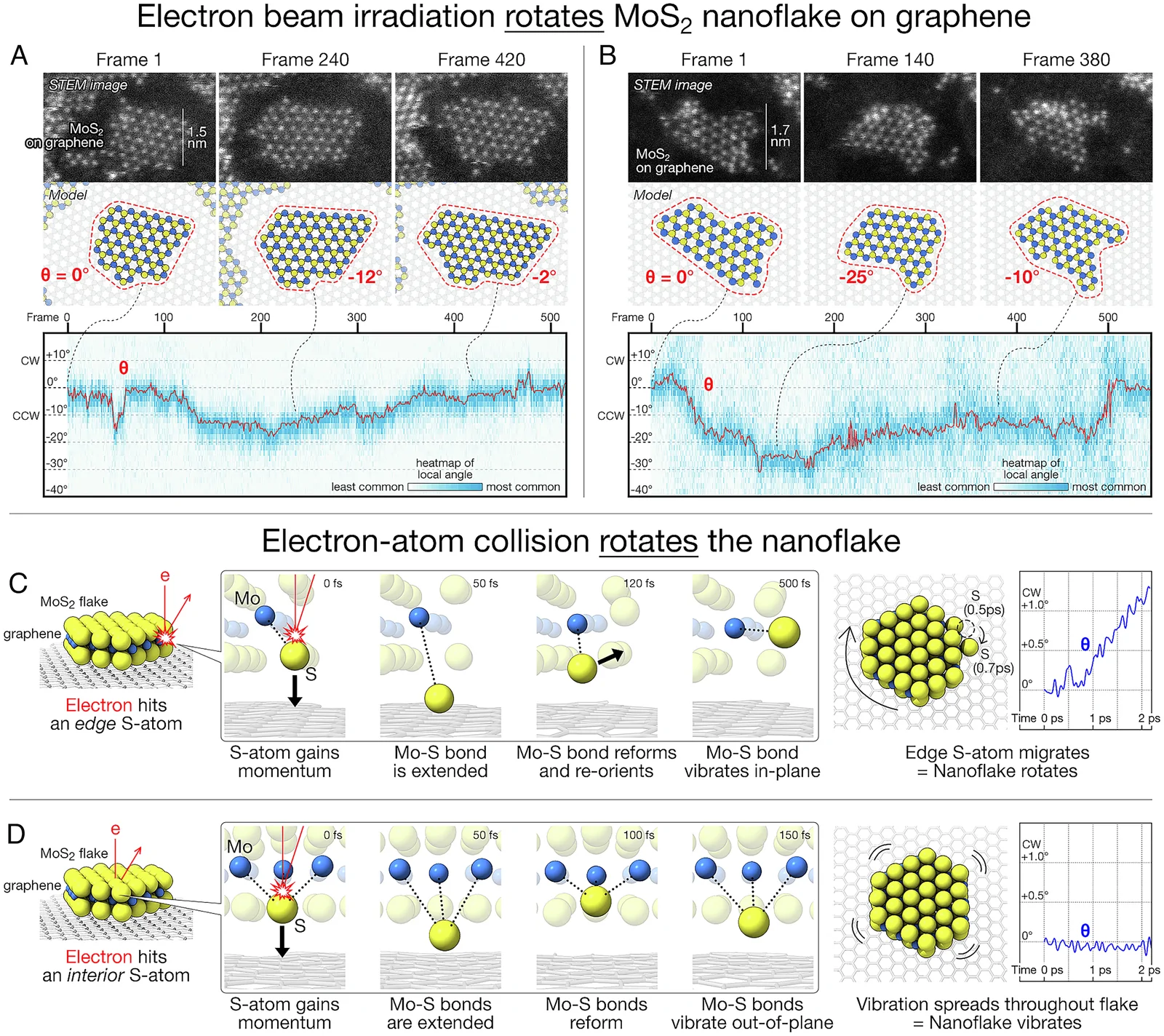
MoS_2_ nanoflake on graphene rotates in-plane due to electron irradiation. Two cases are given in *A* and *B* showing time snapshots of HAADF-STEM images of a MoS_2_ nanoflake (<2 nm wide) changing its global and local orientation tracked at a rate of 4.7 s per frame (see *Materials and Methods* for experiment details). At each frame, the global nanoflake orientation (θ, red line) tracks the rigid body rotation of the nanoflake, while the local nanoflake orientation (white-cyan heatmap, bin size = 1°) tracks the distribution of Mo–Mo nearest neighbor angles in the nanoflake. The orientation is determined relative to the initial orientation at t = 0. Panels *C* and *D* present the computed dynamics, showing the mechanism of how elastic electron–atom collision at the nanoflake edge leads to in-plane rotation of the nanoflake (θ, blue line), while the same collision occurring at the nanoflake interior leads to vibrational excitation of the nanoflake (see *Materials and Methods* for calculation details). CW = Clockwise; CCW = Counterclockwise.

**Fig. 2.**
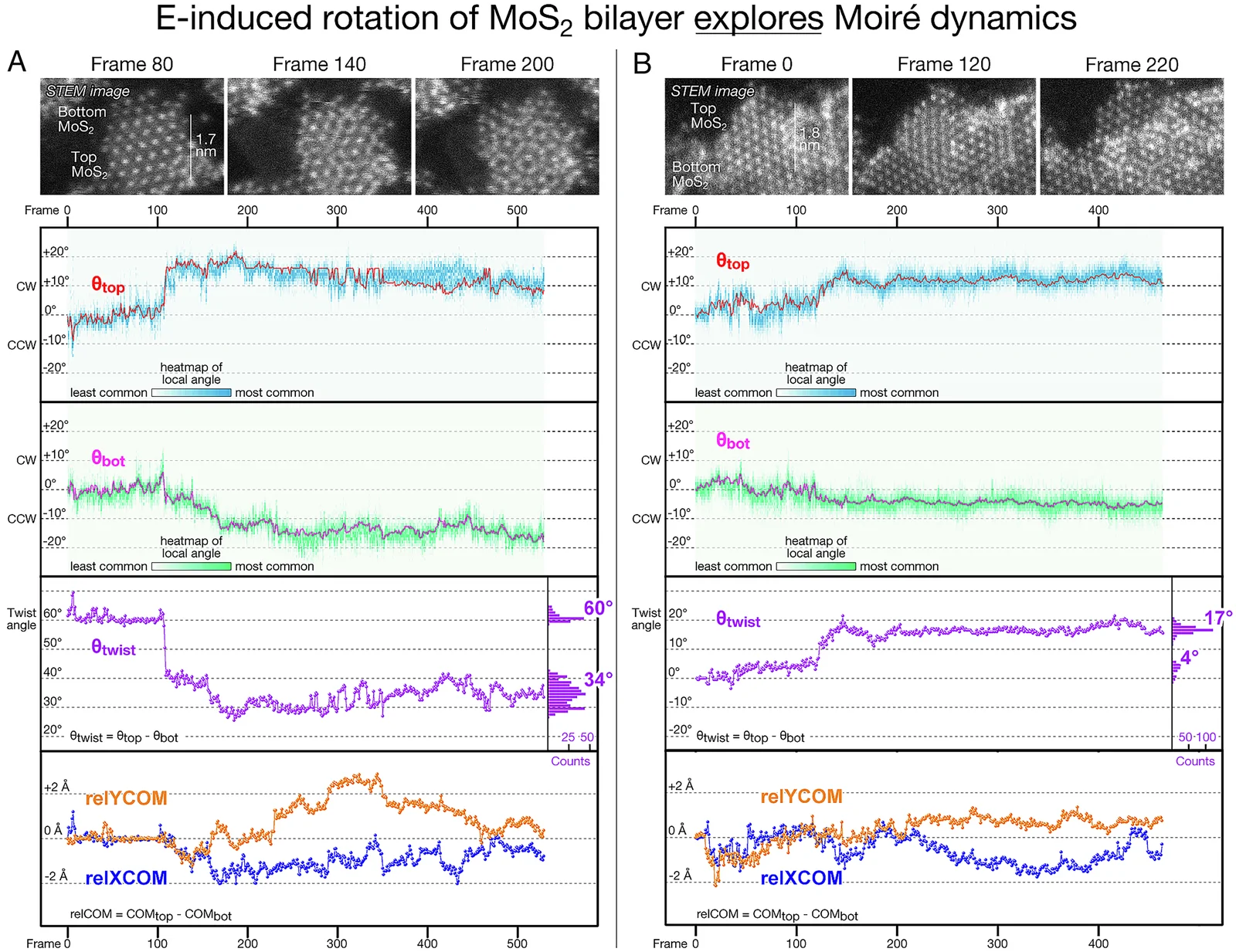
Dynamics of a moiré superlattice observed with atomic resolution. Panels *A* and *B* show two cases of direct tracking of relative orientation (θ_twist_) and relative position (relCOM) between a MoS_2_ nanoflake above another MoS_2_ nanoflake. This is accomplished by tracking both the global and local orientation of both the *Top* and *Bottom* nanoflake simultaneously, shown respectively as a red line (θ_top_) on the white-cyan heatmap for the *Top* nanoflake and a pink line (θ_bot_) on the white-green heatmap for the *Bottom* nanoflake. Relative orientation between the nanoflakes is shown by θ_twist_, where θ_twist_ = θ_top_ – θ_bot_ (violet line) and the corresponding histogram (bin size = 1°), while the relative location between the nanoflakes is shown by relCOM, where relCOM = COM_top_ – COM_bot_ (blue line for translation along x-axis and orange for y-axis). CW = Clockwise; CCW = Counterclockwise.

We prepared and imaged the nanoscale moiré superlattices of MoS_2_ by Electrospray Ion Beam Deposition (ESIBD) (31–33) and High Angle Annular Dark Field Scanning Transmission Electron Microscopy (HAADF-STEM). We chose MoS_2_ as our model system as we were able to prepare nanoflakes (1 to 2 nm in diameter) of monolayer and bilayer MoS_2_ on graphene, critical in ensuring ripple-free moiré domains needed for real-time atomic tracking and θ_twist_ manipulation. Following our previous work (33), we generated monolayer and bilayer MoS_2_ nanoflakes on graphene by depositing mass-selected MoS-oligomer ions onto the graphene held at room temperature in high vacuum (10^−7^ mbar), without any postdeposition annealing. We confirmed the formation of these MoS_2_ nanoflakes by atomically resolved STEM imaging with 60 keV electrons, known to minimize formation of vacancy defects in two-dimensional materials (34). Remarkably, under continuous imaging by the 60 keV electrons, we observed the MoS_2_ nanoflakes to translate and rotate when oriented horizontally (Fig. 1) and to bend and tilt when oriented vertically (*SI Appendix*, Fig. S1). The limited bending and rigid tilting observed of the vertical nanoflakes demonstrate their out-of-plane rigidity, evidencing the resistance of nano-sized flakes to rippling and folding. Moreover, the electron-induced motions of the nanoflakes were noted to be similar to the previously reported amorphous-to-crystalline transition of MoS_2_ films due to 60 keV electron irradiation (35). We subsequently took advantage of these electron-induced dynamics to excite interlayer translations and rotations in the bilayer MoS_2_ nanoflakes, thereby allowing interlayer mobility in moiré superlattices to be observed at atomic scales in real time (Fig. 2).

First, we highlight the dynamics induced by the incident 60 keV electrons on single-layer MoS_2_ nanoflakes on graphene. Fig. 1 presents two representative cases of a MoS_2_ nanoflake continuously observed for ~40 min tracked at a rate of 4.7 s per frame (*SI Appendix*, Fig. S2 and Movies S1 and S2), where the MoS_2_ nanoflake was observed to rotate in-plane and translate in-plane under continuous electron irradiation. Analyzing the dynamics by tracking every Mo-atom in the nanoflake at each frame reveals that the nanoflake rotates as much as 30° (Fig. 1), and translates as much as 7 Å without preferred direction, traversing an area as wide as 13 Å^2^ (*SI Appendix*, Fig. S3). We attribute these extensive rotations and translations to the extremely low computed barriers for rotation (0.02 eV) and translation (0.04 eV) of the nanoflake on graphene, understood as a result of incommensurate contact between the MoS_2_ nanoflake and the underlying graphene. We note that the MoS_2_-graphene nanocontact is always incommensurate due to the different lattice parameters of MoS_2_ and graphene, yielding a flat potential landscape (*SI Appendix*, Fig. S4). Consequently these extensive MoS_2_-on-graphene translations and rotations highlight the prospect for a focused beam of electrons or dressed electrons [e.g., chiral electrons (36)] to control the position and orientation of nanostructures on surface, similar to the use of scanning probe microscopy tip to manipulate atoms and molecules on surface (37).

Modeling of the electron-induced dynamics reveals elastic electron–atom collision as the cause of the observed dynamics. Using ab initio molecular dynamics (MD), we simulated the dynamics initiated by a 60 keV electron colliding elastically and inelastically with an MoS_2_ nanoflake, and found that only the elastic collision leads to outcomes consistent with the experiment. Our results thereby rule out electronic excitation (38) (i.e., transiently ionized MoS_2_ nanoflake) as the cause of the observed dynamics in experiments.

Our MD calculations provide plausible mechanisms behind the observed dynamics by demonstrating conversion of z-axis momentum gained from elastic electron–atom collision into xy-plane nanoflake motion. Fig. 1 *C* and *D* present two representative dynamics initiated by adding a downward momentum to an S-atom within the nanoflake with its magnitude estimated from the relativistic kinematics for elastic electron–atom collisions (39, 40). Momentum added to edge S-atoms leads to nanoflake rotation due to the edge S-atom reorienting and migrating to produce a torque that rotates the nanoflake (Fig. 1*C*). Consistent with the MD calculation, such edge structure reconstruction was observed experimentally (Fig. 1 *A* and *B*), and may be one of the causes of the nanoflake rotation. By contrast, momentum added to an interior S-atom only leads to nanoflake vibration without significant rotation, because the interior S-atoms are unable to migrate away from their initial positions (Fig. 1*D*). While these two MD calculations provide evidence for the plausible energy-conversion pathway to rotation and vibration in the picosecond timescale, we expect at the nanosecond timescale the nanoflake to equipartition the energy gained from the electron–atom collision to all its degrees-of-freedom, which include translation, rotation, and vibration. Our MD calculations thus provide a mechanistic framework in understanding electron-induced motion of nanostructures on surface. We anticipate that the electron beam energy and electron flux are two additional parameters to achieve controlled nanostructure manipulation by electron beam, whereby the beam energy would dictate the amount of energy transferred from the incident electron onto the nanostructure, while the electron flux would dictate the frequency of such energy transfer.

Taking advantage of these electron–atom interactions, we induced motions between layers of a moiré superlattice to observe dynamical signatures of high and low interlayer mobility at atomic resolution. Fig. 2 presents two representative cases of a bilayer MoS_2_ nanoflake with an interlayer contact area of ~2.4 nm^2^ (Fig. 2*A*) and ~4.9 nm^2^ (Fig. 2*B*) observed for ~40 min at a rate of 4.7 s per frame (*SI Appendix*, Fig. S2 and Movies S3 and S4). In both cases, we again tracked every top-layer and bottom-layer Mo-atom in every frame to follow the dynamics of interlayer rotation (twisting) and interlayer translation (sliding), here denoted by changes in twist angle (θ_twist_) and relative center-of-mass (relCOM) respectively. As shown by the images (Fig. 2) and their profiles (*SI Appendix*, Fig. S5), both cases were observed initially with a commensurate interlayer contact that changed into an incommensurate contact, namely from the AB′-stack to a moiré in Fig. 2*A*, and in Fig. 2*B*, from the AA-stack to a moiré. These two cases reveal the characteristic twisting and sliding of commensurate and incommensurate contacts, thus providing atomic-level insight into the origins of high and low interlayer mobility in moiré superlattices. We note that we analyzed only cases exhibiting interlayer rotation on the experimental timescale, confirming sufficient nanoflake mobility without persistent defect pinning. Atomic-resolution imaging of the Mo and S lattices further confirms that MoS_2_–MoS_2_ contact dominates the interface.

**Fig. 3.**
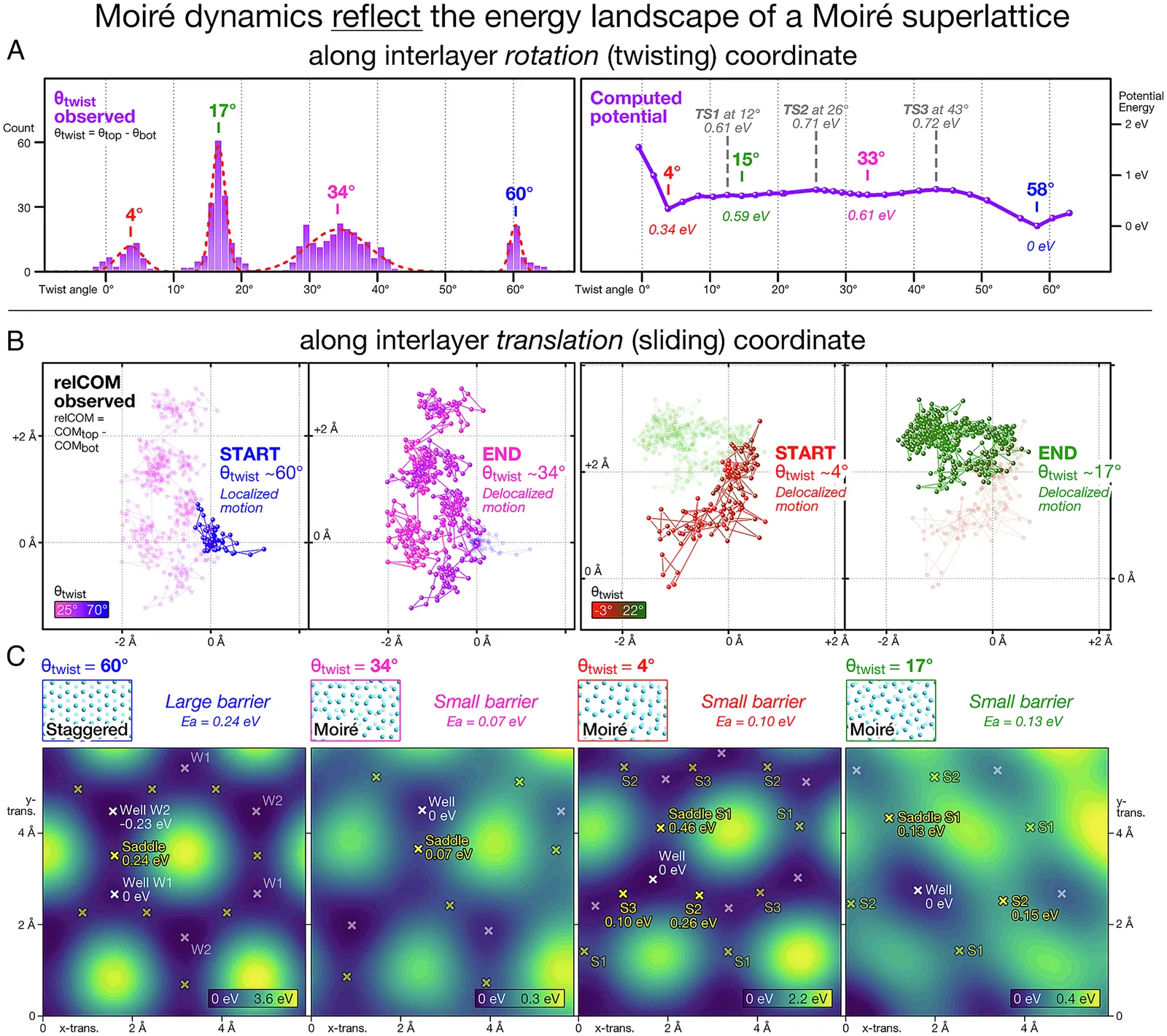
Moiré dynamics reflect the energy landscape of interlayer twisting and sliding. The twist angle (θ_twist_) and relative translation (relCOM) tracked between two MoS_2_ nanoflakes reflect the energy landscape along the interlayer rotation (i.e., twisting) and interlayer translation (i.e., sliding) degree-of-freedoms. Panel *A* presents the observed and computed stable θ_twist_ states. The observed θ_twist_ distribution (bin size = 1°) is fitted with Gaussian curves (red dashed line) shown with their respective peak positions, while the computed potential along θ_twist_ is shown with every potential well and transition state marked with its respective energy. Panels *B* and *C* present the observed relCOM on xy-plane for the two cases in Fig. 2 together with computed potential along xy-translation at various θ_twist_, each shown with all saddle points (yellow cross) and potential wells (white cross) marked.

The first dynamical signature of high interlayer mobility in moiré superlattices is the anomalous stability of incommensurate contact structures due to entropic stabilization. We observed, in both cases (Fig. 2), a transition from a commensurate structure (θ_twist_ = 60°) or near-commensurate structure (θ_twist_ = 4°) into incommensurate structures (θ_twist_ = 17° or 34°), without returning. Together, these dynamics yield θ_twist_ distribution with peaks at 4°, 17°, 34°, and 60°, confirmed as four potential wells at 4°, 15°, 33°, and 58° on the computed energy landscape along θ_twist_ (Fig. 3*A*). The energy landscape thus explains the large, sudden change in θ_twist_ (e.g., frame 100 to 110 of Fig. 2*A*) as a successful barrier crossing to a neighboring well, and the occasional large spikes in θ_twist_ (e.g., frame 0 to 10 of Fig. 2*A*) as a failed barrier crossing that returns the system back to its initial well. Notably, the computed energy landscape shows that the commensurate states near 0° and 60° to be energetic minima, while the incommensurate states near 15° and 35° are situated at a near-flat landscape at higher energies, which persists against variations in nanoflake size, edge structure, and defect presence (*SI Appendix*, Fig. S6). We expect these dynamics and energy landscape to provide the atomic-level frameworks to achieve coherent control of moiré superlattice dynamics (38).

Interestingly, the energy landscape also reveals that, in both cases (Fig. 2), the system transitioned from low-energy states (θ_twist_ = 4° or 60°) into high-energy states (θ_twist_ = 17° or 34°). In addition, we also observed multiple independent observations where the system transitioned from these high-energy states to low-energy states (*SI Appendix*, Fig. S7). We point out that direct experimental observation of these high-energy states, despite the presence of low-energy states, suggests a mechanism that allows the system to stay in these high-energy states over experimentally observable timescales. One possible mechanism is entropic stabilization—a stabilization mechanism common in highly disordered solids (41) and proteins (42, 43). The entropic stabilization of the high-energy states is supported by the near-flat energy landscape around high-energy states, evidenced by the wider (nearly two-fold) spread of θ_twist_ distribution for the high-energy states (θ_twist_ = 17° or 34°) when compared to the low-energy states (θ_twist_ = 4° or 60°) (Fig. 3*A* and *SI Appendix*, Fig. S7). The near-flat energy landscape, confirmed by DFT (Fig. 3*A* and *SI Appendix*, Fig. S6), is understood to provide a vast number of near-degenerate microstates that lower the probability for the system to find its way back to the low-energy states. Additionally, in such region, the weak potential energy gradients generate correspondingly weak steering forces (torques) that allow the system to stay in these incommensurate states. We attribute the flatness of the energy landscape to the incommensurate interface largely preserving the sum of attractive and repulsive interactions upon twisting. The entropic stabilization of incommensurate structures due to their near-flat energy landscape shown here serves as one of the key dynamical signatures of high interlayer mobility in moiré superlattices.

Next, we show enhanced sliding at incommensurate interface as another key signature of high interlayer mobility in moiré superlattices. In both cases (Fig. 2), the observed interlayer translation (sliding) dynamics reveal distinct sliding behaviors governed by the commensurability of the interlayer contact. With a commensurate contact (θ_twist_ = 60°), sliding rarely occurs (frame 0 to 110 in relCOM of Fig. 2*A*), whereas with near-commensurate (θ_twist_ = 4°) or incommensurate contacts (θ_twist_ = 17° and 34°), sliding occurs often (frame 110 to 529 in relCOM of Fig. 2*A* and frame 0 to 464 in relCOM of Fig. 2*B*). Visualizing the sliding dynamics on xy-plane establishes the strong effect of the interface commensurability on the area traversed by the sliding motions (Fig. 3*B*). The commensurate interface (θ_twist_ = 60°) gives highly localized motions within a small area of 0.6 Å^2^. In contrast, the near-commensurate (θ_twist_ = 4°) and incommensurate interfaces (θ_twist_ = 17° and 34°) exhibit highly delocalized motions over areas as large as 3.0 Å^2^, 2.8 Å^2^, and 6.9 Å^2^, respectively, yielding up to 12-fold enhancement relative to the commensurate case.

The distinct sliding dynamics observed at various contact geometries are well understood by the computed energy landscapes of interlayer sliding at various θ_twist_ (Fig. 3*C*). Our calculations yield barriers consistent with the observed trend for the area traversed by the sliding dynamics. The near-commensurate (θ_twist_ = 4°) and incommensurate contacts (θ_twist_ = 17° and 34°) lead to barriers as low as 0.10 eV, 0.13 eV, and 0.07 eV respectively, which are 45 to 70% lower than the 0.24 eV barrier from the commensurate contact (θ_twist_ = 60°) (Note: the barrier at θ_twist_ = 60° is computed from the observed AB′-stack structure that corresponds to the W1 well in Fig. 3*C*). We attribute the barrier reduction to the flattening of energy landscape as the interlayer contact gets increasingly incommensurate, as a result of the incommensurate interface largely preserving the sum of attractive and repulsive interactions upon sliding. The stark 12-fold difference in area traversable by the sliding dynamics at commensurate and incommensurate interfaces therefore serves as evidence of mobility switching in moiré superlattices.

**Fig. 4.**
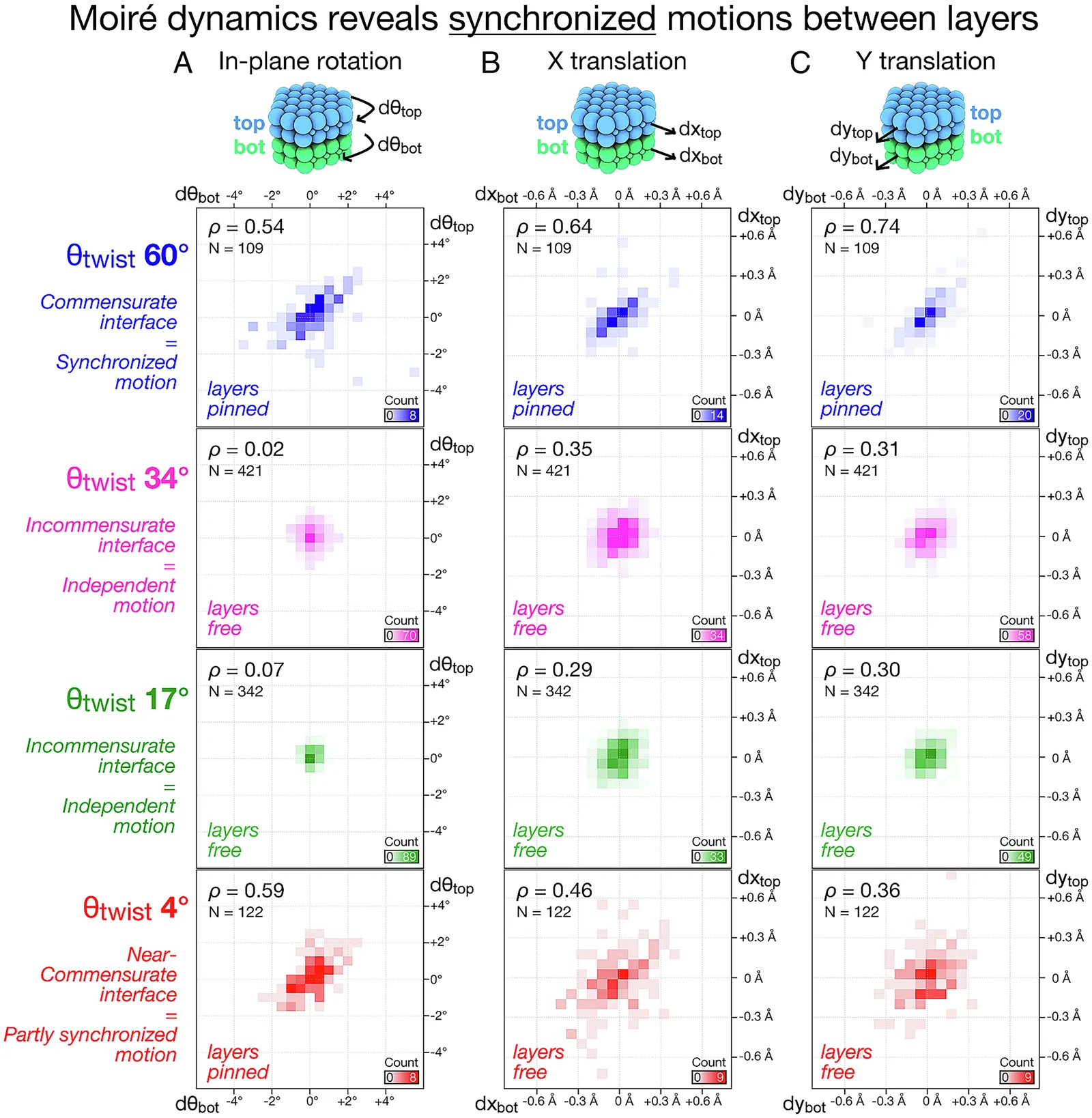
Synchronized rotation and translation between layers reveals interlayer pinning. Directional and magnitudinal correlations at various contact interfaces dictated by twist angles (θ_twist_) are shown between the angular displacements of the *Top* (dθ_top_) and *Bottom* (dθ_bot_) nanoflakes in *A*, between the translation along the x-axis of the *Top* (dX_top_) and *Bottom* (dX_bot_) nanoflakes in *B*, and between the translation along the y-axis of the *Top* (dY_top_) and *Bottom* (dY_bot_) nanoflakes in *C*. In every panel, a 2D-histogram (bin size: 0.5° in *A* and 0.075 Å in *B* and *C*) is shown together with the number of observation (N), whereby the strength of correlation is measured by the Spearman’s correlation (*ρ*) in which the value of 0 indicates no monotonic relationship between the two variables while 1 indicates perfect positive monotonic relationship between two variables (i.e., when one variable increases, another variable consistently increases).

The second evidence of mobility switching in moiré superlattices is given by the observation of synchronized atomic motions in the commensurate interface and independent motions in the incommensurate interface. To demonstrate this, we correlate top-layer rotations to bottom-layer rotations (Fig. 4*A*) as well as top-layer translations to bottom-layer translations (Fig. 4 *B* and *C*) to obtain respective rotational and translational correlation strengths (ρ), which indicate the degree of orientational and positional pinning between layers. High ρ-values indicate interlayer pinning, where both layers move as a single rigid body; whereas low ρ-values indicate interlayer depinning, where the layers move independently of one another, signifying absence of energy dissipation between the layers, a hallmark of the superlubricity-like effect.

The ρ-values obtained from experiments are found to be attributable to the lateral forces exerted between layers. For every contact geometry, we show the ρ-value trends to be consistent with the trends of the maximum restoring torque (*τ*_max_) and restoring force (F_max_) along the minimum energy path for twisting (Fig. 3*A*) and sliding (Fig. 3*C*). In case of rotations (Fig. 4*A*), our results show that synchronized rotations (ρ > 0.5) are caused by large *τ*_max_ (72 meV/degree for θ_twist_ = 60° and 62 meV/degree for θ_twist_ = 4°), whereas independent rotations (ρ < 0.5) are caused by small *τ*_max_ (18 meV/degree for θ_twist_ = 17° and 34°). Similarly, in case of translations (Fig. 4 *B* and *C*), our results show that synchronized translations (ρ > 0.5) are caused by large F_max_ (837 pN for θ_twist_ = 60°), whereas independent translations (ρ < 0.5) are caused by small F_max_ (316 pN, 185 pN, 88 pN for θ_twist_ = 4°, 17°, and 34° respectively), which are within the regime of forces measured (20, 21) and calculated (28) in superlubric systems.

Our results lead to the identification of interlayer pinning profile that depends on contact geometry, while also showing that the moiré superlattice accesses a greater level of interlayer mobility as the contact incommensurability increases. At one extreme, the incommensurate contacts (θ_twist_ = 17° and 34°) cause full orientational and positional depinning, resulting in high interlayer mobility. At another extreme, the commensurate contact (θ_twist_ = 60°) causes full orientational and positional pinning that lock the moiré superlattice into one rigid body. Between these extremes, the near-commensurate contact (θ_twist_ = 4°) causes an interesting combination of orientational pinning and positional depinning that allow interlayer sliding, but not twisting. These findings underscore the rich influence of contact interface in switching interlayer mobility on or off in moiré superlattices.

Through atomically resolved imaging, our work reveals the key role of the interface commensurability in switching interlayer mobility on or off. The observed decrease in contact commensurability, which leads to increased mobility, thus demonstrates the central tenet of the superlubricity-like effect: Incommensurate interface reduces the motional energy barriers because the sum of attractive and repulsive interfacial forces remains unchanged during interlayer motion. This idea is similar to the notion that energy barriers of chemical reactions decrease if the energy cost to break a bond is concurrently compensated by an energy release from a bond formation event (44).

As an outlook, the interlayer mobility switching observed for the nanoscale moiré superlattices here provides new strategies in realizing macroscopic superlubric surfaces and dynamic twistronic devices. To realize the former, the apparent rigidity of nano-sized flakes leads us to propose tiling nanoflakes on surfaces, similar to ceramic tiles on floors (45), to bypass issues caused by rippling and folding of large-scale layered materials (30). Assembling nanoflakes uniform in size and shape on atomically flat surfaces would give surfaces tiled with randomly oriented nanoflakes. Consequently, these randomly oriented nanoflakes would guarantee that the contact between the two tiled surfaces to be incommensurate and superlubric. On the other hand, in realizing dynamic twistronics devices, the dynamics of nanoscale moiré superlattices observed here, such as the interlayer pinning with commensurate contact or interlayer depinning with incommensurate contact, allow rich engineering opportunities for devices made by stacking the nanoflakes, similar to the nanocoins (46) or the “Tower of Hanoi” (47). First, either side of a nanoflake could be atomically modified into Janus-type (48) or into QR-like coded pattern (49) to tailor its orientational and positional pinning profile with its top and bottom neighbors, ranging from completely superlubric to lock-and-key interactions. Second, a custom stacking sequence of nanoflakes from diverse two-dimensional materials (7) (e.g., metallic, semiconducting, magnetic, insulating, etc.) could be realized similar to sequence-specific polymers (50) or to heterojunction transistors (51) to achieve desired transport properties between the nanoflakes. Third, the edge of the nanoflakes could be conjugated to covalent staples (52), photoredox agents (53), or DNA- (54) or protein-based nanomotors (55) that allow active control of the device by external electric, light, or chemical stimuli as nanoscale rotors or gears. Combining these elements would undoubtedly require bridging rich ideas from the field of chemical synthesis, click chemistry, supramolecular chemistry, and biotechnology into the new frontier of nanoscale moiré superlattices.

## Materials and Methods

### Materials.

Ammonium tetrathiomolybdate ((NH_4_)_2_MoS_4_, 99.97%, Catalog#: 323446, CAS: 15060-55-6) and ammonium persulfate ((NH_4_)_2_S_2_O_8_, 98%, Catalog#: 248614, CAS: 7727-54-0) were purchased from Sigma Aldrich. PMMA-coated CVD-grown graphene on a Cu-foil was purchased from Graphenea. Holey SiN support grid was purchased from Norcada, Inc. (Catalog#: NH050D05).

### Methods.

#### Single-layer graphene substrate preparation.

The graphene substrate preparation followed the previously reported procedure (33, 56). In brief, the PMMA-coated graphene on Cu-foil was etched with a dilute ammonium persulfate solution (8 g in 100 mL water) to dissolve the Cu-foil and release the PMMA-coated graphene onto the liquid surface. Following the transfer of the PMMA-coated graphene onto a Pt-coated SiN grid (~10 nm thick of Pt) and the removal of the PMMA by annealing the grid in ambient atmosphere at 300 °C, the graphene-on-SiN grid was subsequently transferred into the sample holder in a vacuum loadlock for ion beam deposition.

#### Ion beam deposition on graphene.

Using the home-built electrospray ion beam deposition (ESIBD) setup (32, 57), ions of MoS-oligomers [(MoS_3_)_n_SH^−1^, n ≥ 4] were obtained from electrospray ionization, mass-selected, and deposited intact with 3 eV of landing energy on the graphene-on-SiN grid held at room temperature in a high vacuum chamber (~10^−7^ mbar). The coverage of the molecule on surface was monitored by integrating the ion current arriving on the grid. Following the deposition, the chamber was vented with dry nitrogen and the grid was transferred in ambient atmosphere into the transmission electron microscope.

#### Direct imaging of samples by transmission electron microscopy.

Scanning transmission electron microscopy (STEM) was used to observe the result of MoS-ions deposition on the graphene-on-SiN grid. As reported previously (33), the deposited MoS-oligomers formed MoS-nanoribbons on which MoS-nanoflakes, the focus of present work, were occasionally found nearby the parent ribbon or found on top of a nanoribbon. The STEM was operated with a 60 keV electron beam energy in HAADF (High Angle Annular Dark Field) mode, whereby only electrons deflected at high angles were detected. STEM-HAADF imaging was performed with an aberration-corrected JEOL ARM200F STEM instrument, equipped with a cold-field emission gun and a spherical aberration corrector (DCOR, CEOS GmbH). Single HAADF-STEM image was acquired by JEOL ADF detector with a convergent semiangle of 33.5 mrad and collection semiangles of 56 to 234 mrad with 16 μs/pixel dwell time, while time-series of HAADF-STEM images were acquired with 3 μs/pixel dwell time and 1,500 μs flyback time (except for *SI Appendix*, Fig. S1, where the flyback time of 3,000 μs was used for the case of MoS_2_ nanoflake bending).

#### Dose measurement.

Electron dose was measured by the AXON Dose system (Protochips, Inc.) (58), which includes the AXON Dose calibration software module and the AXON Dose TEM calibration holder with an integrated Faraday cup at the tip position. The system was used to measure incident electron current and thereby measure electron dose, cumulative dose, and dose rate across a sample as a function of time and location. The dose given in this work was calculated by averaging the dose rate in areas as big as 9.7 × 9.7 nm^2^ measured up to 13 min. As a result, every frame in the recorded movie data was taken with a dose rate of 6.0 × 10^4^ electrons/Å^2^ for the cases in Fig. 1 *A* and *B* and 2*A* and 1.4 × 10^5^ electrons/Å^2^ for the cases in Fig. 2*B*.

#### STEM-HAADF image analysis.

Prior to analysis, all images were calibrated by scaling the measured lattice parameters of single-layer graphene from the STEM-HAADF images to the known value of 2.46 Å in literature (59). Custom python programs using the ncempy package (60) were written to read the STEM-HAADF movies, remove the thermal drift, and track the location of every Mo-atom in the nanoflake at every frame to determine its rotation and translation.

To determine the nanoflake translation, we approximated the center of mass (COM) of the nanoflake on the first frame of the movie data and identified the few closest Mo-atoms to the COM coordinate, termed MoCOM-atoms. The COM in subsequent frames was computed from the coordinates of tracked MoCOM-atoms, which revealed where the COM was on every frame.

To determine the nanoflake rotation, we determined on every frame both “local” and “global” nanoflake angles whose changes were both validated by the Fourier-transform analysis of the Mo-lattices. To determine the local angle, we computed on every frame the absolute angle made by every pair of Mo–Mo nearest neighbor against the positive x-axis and then used the hexagonal symmetry of the Mo-lattice in MoS_2_ to fold every computed angle to within 0° and 60°, thereby giving a distribution of local Mo–Mo angles in a nanoflake at a given frame (presented as a 2D histogram/heatmap in Figs. 1 and 2). To determine the global angle, we performed on every frame a least-square fit of an ideal hexagonal Mo-lattice with 3.16 Å lattice parameter to the detected Mo-atoms in the nanoflake to yield an angle of a rigid body fit at a given frame (presented as a line in Figs. 1 and 2).

The extent of nanoflake translation was determined by the area of the alpha shape obtained from the recorded (x,y) coordinates (61). The alpha shape determination was performed using the “alphashape” package (https://pypi.org/project/alphashape/) in Python.

#### Ab initio calculations.

DFT calculations were performed to model the experimental observations. Following previously known parameters that reproduced the experiment well (33), the Vienna Ab-initio Simulation Package (VASP, version 5.4.4) (62, 63) implementing the plane-wave basis sets was employed with a projection-augmented wave function (PAW) method (64, 65), a cut-off energy of 400 eV, the Perdew–Burke–Ernzerhof (PBE) functional (66), and the Grimme’s DFT-D3 approach (67). MoS_2_ nanoflake on graphene was modeled with a hexagonal-shape Mo_27_S_54_ nanoflake on a ~40 × 40 Å^2^ single-layer graphene slab with a ~20 Å vacuum space above the slab. Interactions between MoS_2_ nanoflakes were modeled with a MoS_2_ nanoflake on a ~40 × 40 Å^2^ single-layer MoS_2_ slab with a ~20 Å vacuum space above the slab, whereby the nanoflakes used were similar in size to the ones observed in the experiment, such as Mo_27_S_54_ nanoflake (d ~ 1.5 nm) for the Fig. 2*A* case and Mo_75_S_150_ (d ~ 2.8 nm) for the Fig. 2*B* case. Structures were relaxed until every force acting on every atom was below 0.02 eV/Å.

We modeled two possible excitations due to electron–atom collision in a MoS_2_ nanoflake, namely electronic excitation (i.e., creation of a hole in the valence shell of Mo- or S-atom) or electron-to-atom momentum transfer. The effect of electron-to-atom momentum transfer from electron–atom collision in a MoS_2_ nanoflake on graphene was modeled by MD calculations implemented in VASP with a time-step of 0.5 fs as a microcanonical ensemble. First, every atom was initialized with random velocities sampled from the Boltzmann distribution at 300 K. Then a downward velocity corresponding to specific kinetic energy was added to a specific single Mo- or S-atom to simulate the effect of electron–atom collision and the system was evolved for several picoseconds to examine the effect of the added velocity on the overall dynamics of the nanoflake. The amount of kinetic energy added to atoms assumed an elastic collision process between a 60 keV electron and either a Mo- or an S-atom, which following the previously established relativistic kinematics calculations (39, 40) gave a maximum possible energy transfer of 4.34 eV for S-atom and 1.45 eV for Mo-atom. Alternatively, the effect of electronic excitation on the MoS_2_ nanoflake dynamics was examined using the Impulsive Two-State (I2S) model (68, 69) which also employed the MD calculations implemented in VASP. However, the result revealed no significant nanoflake dynamics due to hole delocalization in MoS_2_.

The potential energy landscape along the interlayer rotation (twisting) coordinate was obtained by performing nudged elastic band (NEB) calculations to determine the minimum energy pathway along the twisting coordinate between the relaxed structures. All NEB calculations were performed with 0.02 eV/Å force convergence criteria with the climbing-image (CI) mode activated to determine the saddle points along between the potential wells (70). The potential energy landscape along the interlayer translation (sliding) coordinate was obtained by performing single point calculations of variously rotated and translated MoS_2_ nanoflake on a MoS_2_ slab. Mo_27_S_54_ was used to model the case in Fig. 2*A* while Mo_75_S_150_ was used to model the case in Fig. 2*B*. Potential energies were normalized based on their contact area to allow comparison between results obtained from Mo_27_S_54_ and those from Mo_75_S_150_.

## Supplementary Material

Appendix 01 (PDF)

Movie S1. **First observation case for single layer MoS_2_ nanoflake on graphene**. The recorded data was taken over 517 frames (~40 minutes) at a rate of 4.68 seconds per frame with acquisition breaks shown in Fig S2A. Scale: 100 pixels = 6.89 Å. The movie shows the frames at 15 frames per second.

Movie S2. **Second observation case for single layer MoS_2_ nanoflake on graphene**. The recorded data was taken over 549 frames (~42 minutes) at a rate of 4.68 seconds per frame with acquisition breaks shown in Fig S2B. Scale: 100 pixels = 6.63 Å. The movie shows the frames at 15 frames per second.

Movie S3. **First observation case for bilayer MoS_2_ nanoflake on graphene**. The recorded data was taken over 530 frames (~41 minutes) at a rate of 4.68 seconds per frame with acquisition breaks shown in Fig S2C. Scale: 100 pixels = 6.89 Å. The movie shows the frames at 15 frames per second.

Movie S4. **Second observation case for bilayer MoS_2_ nanoflake on graphene**. The recorded data was taken over 464 frames (~36 minutes) at a rate of 4.68 seconds per frame with acquisition breaks shown in Fig S2D. Scale: 100 pixels = 10.56 Å. The movie shows the frames at 15 frames per second.

## Data Availability

Study data are included in the article and/or supporting information.
