# Supplementary material for: Interlayer rotation (twisting) switches interlayer mobility in moiré superlattices
Source: Proc Natl Acad Sci U S A. 2026 Sep 22;123(39):e2612453123. doi: 10.1073/pnas.2612453123 (PMC13624630; doi:10.1073/pnas.2612453123)
Supplement: Supplementary file 1 [file pnas.2612453123.sapp.pdf]

Interlayer rotation (twisting) switches interlayer mobility in moiré superlattices

Xuejiao Zhang†*, Jimin Ham†, Vesna Srot, Peter A. van Aken, Kelvin Anggara*
Max Planck Institute for Solid State Research, Heisenbergstrasse 1, Stuttgart, 70569, Germany.

† XZ and JH contributed equally to this work.

***Corresponding authors:**
Xuejiao Zhang (xj.zhang@fkf.mpg.de)
Kelvin Anggara (k.anggara@fkf.mpg.de)

Supplementary Information

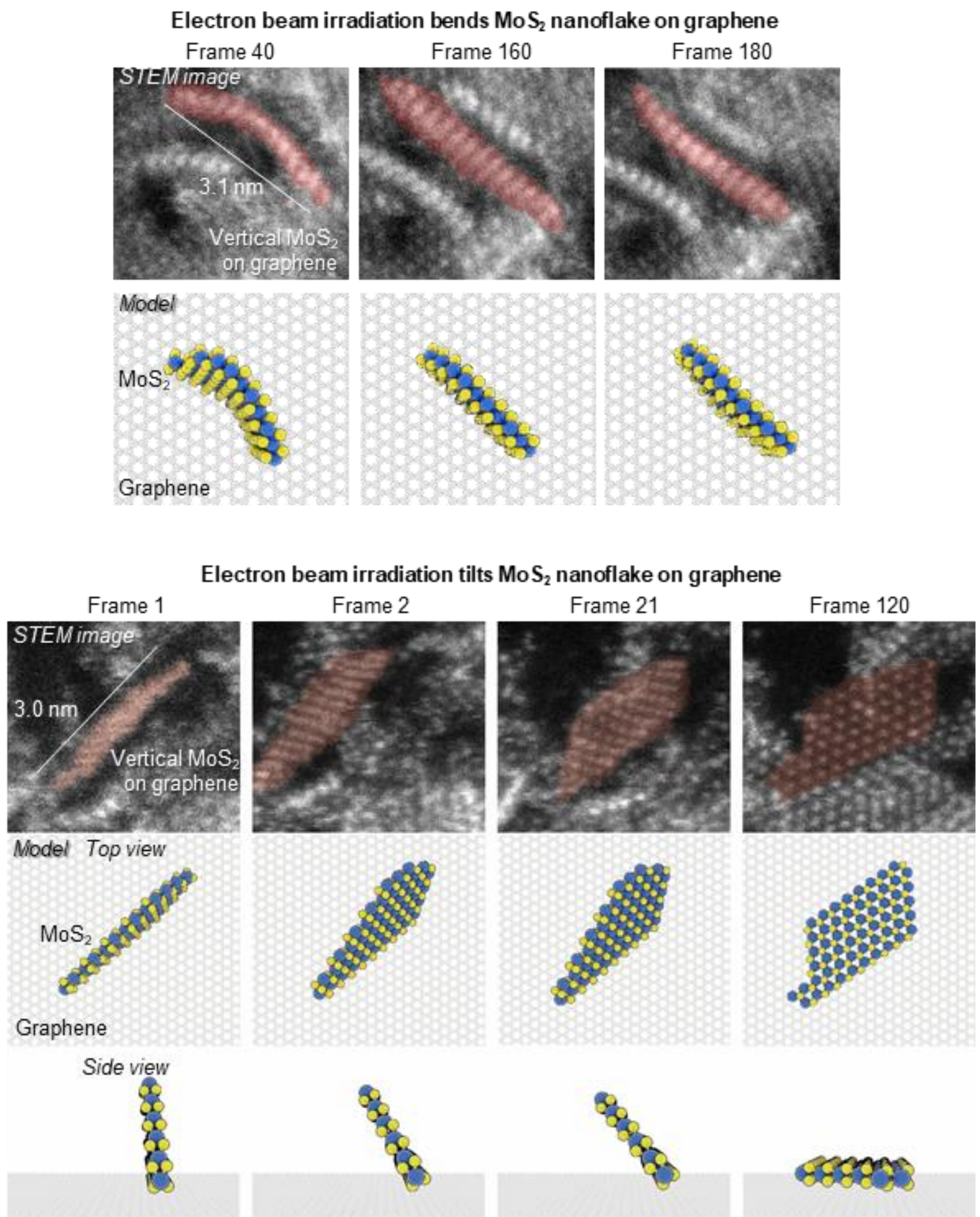

Fig. S1 | Vertical MoS_2 nanoflake on graphene. Upon electron irradiation at 60 keV, the vertical nanoflakes (highlighted in red) were observed to bend or tilt. In the example shown above, the vertical nanoflake was observed to bend transiently as much as ~25° to the left (e.g. frame 40) and ~6° to the right (e.g. frame 180), before returning to its equilibrium state where the nanoflake did not bend at all (e.g. frame 160). On the other hand, the vertical nanoflake was observed to rotate out-of-plane (i.e. tilt) from the vertical state to the horizontal state as a rigid body due to the

observed atomic lattice that agrees well with tilted, rigid nanoflake. The limited bending and rigid tilting demonstrate the out-of-plane rigidity of the nanoflake and thus its resistance toward rippling and folding present in micron-scale layers. Also, we computed the horizontal state to be the most stable state of the two states.

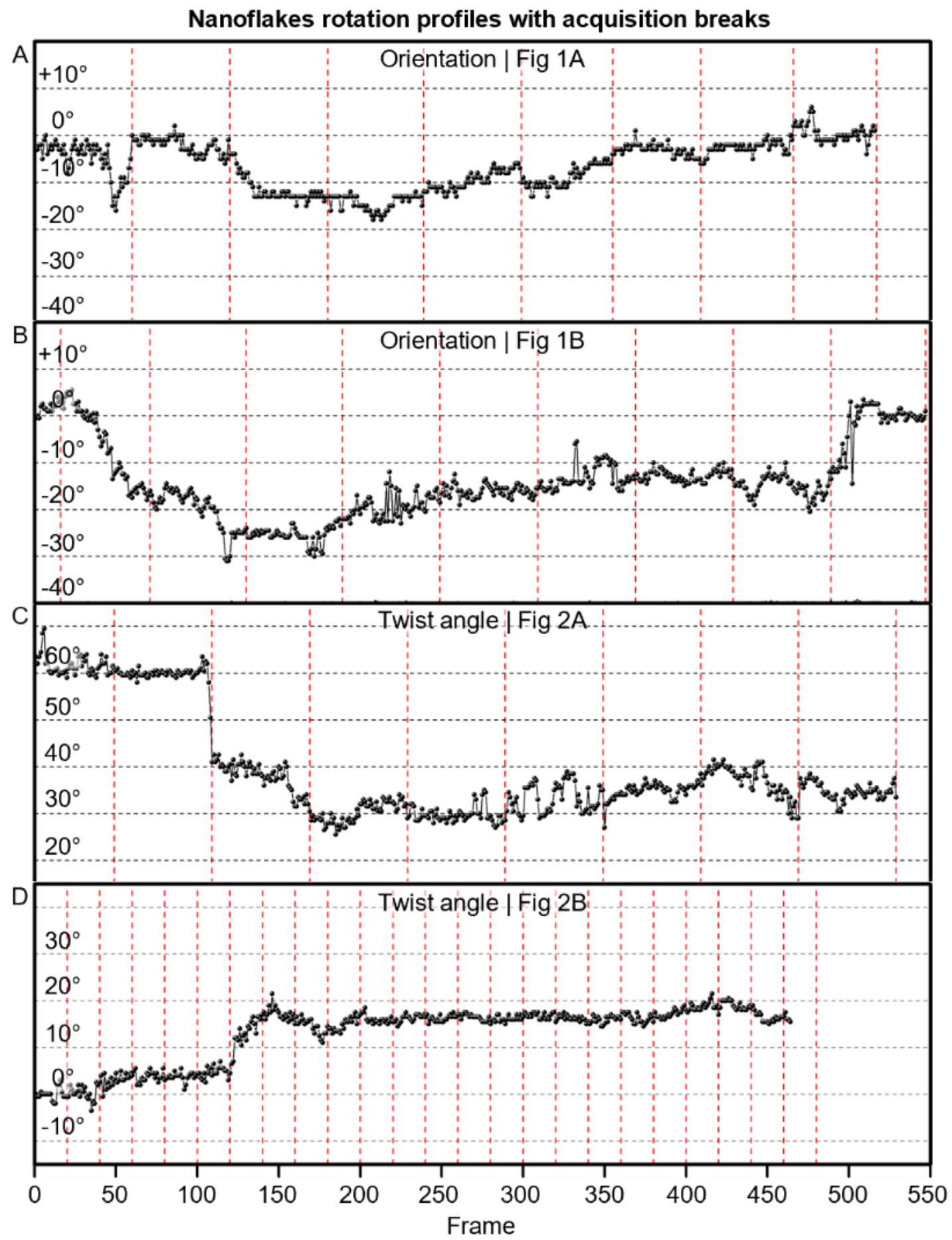

Fig. S2 | Recorded dynamics contain acquisition breaks. The continuous data showing MoS_2 nanoflake dynamics were taken with few acquisition breaks (marked by red dashed line), where the files were manually saved for 2-3 minutes. During this time, the electron beam still continued to irradiate the observation area. From the top, the first and second panels refer to the data from Movie S1 and Movie S2, shown in Fig 1A and Fig 1B, respectively. The third and fourth panels refer to the data from Movie S3 and S4, shown in Fig 2A and Fig 2B respectively.

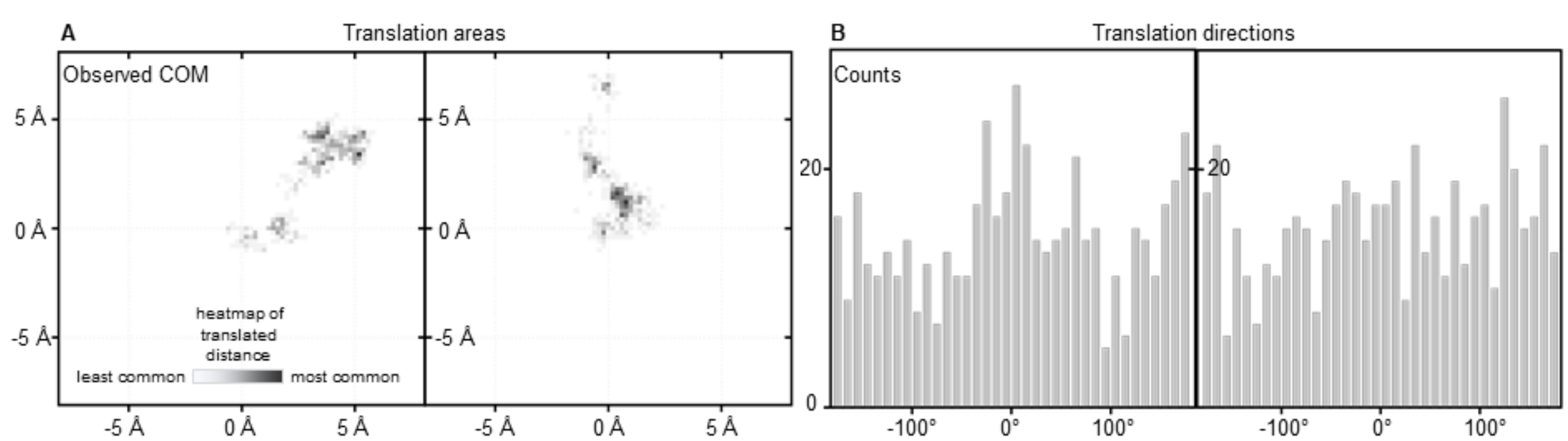

Fig. S3 | Analysis of MoS_2 nanoflake translation on graphene. Panel (A) shows binned translation data on xy-plane (bin size: 0.2 Å), showing the flake traveled over an area as large as 12.3 $Å^2$ for the first observation case (Fig 1A) and 13.4 $Å^2$ for the second case (Fig 1B). Panel B shows that MoS_2 nanoflake on graphene exhibits no directional preference in its translational dynamics.

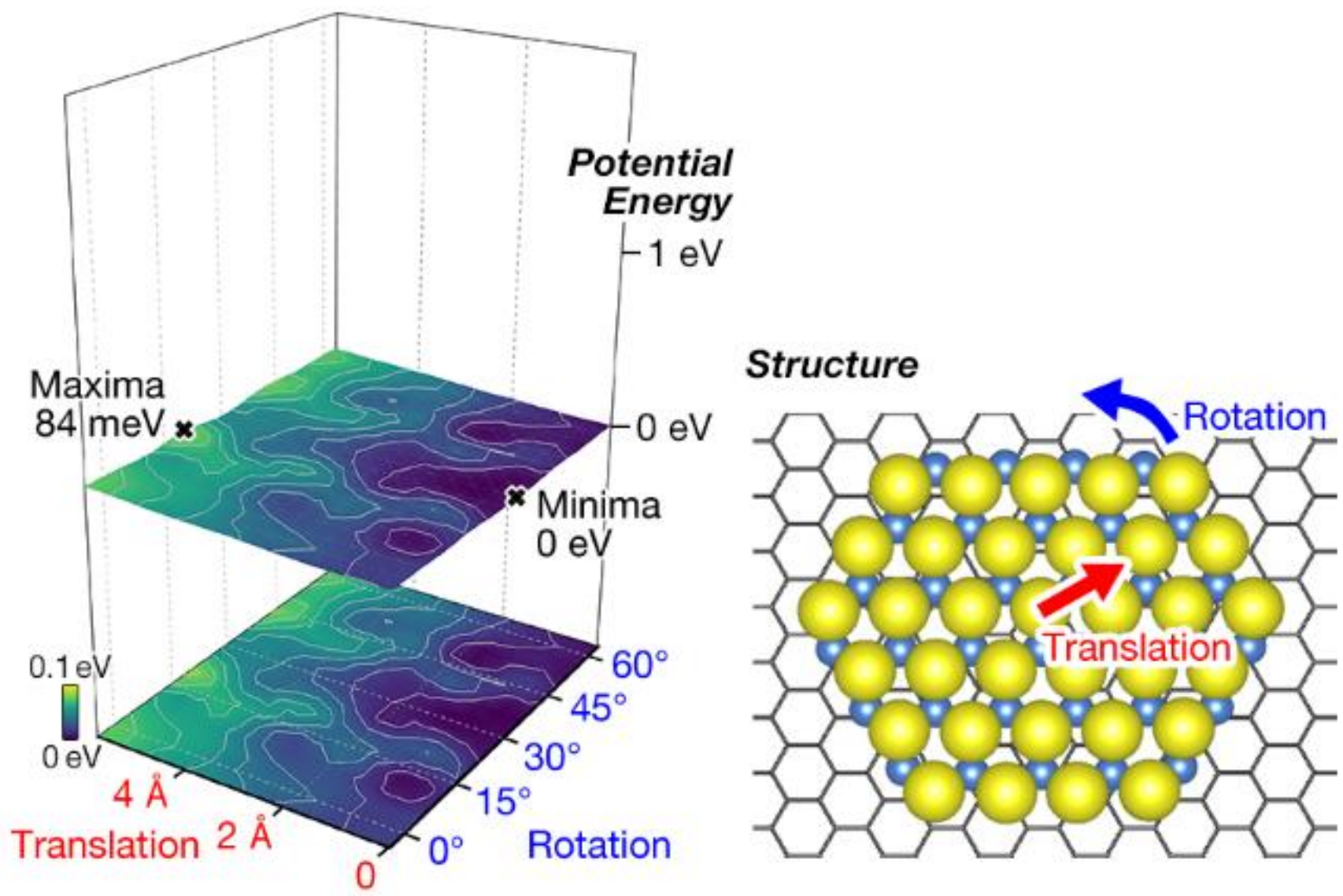

Fig. S4 | Computed potential energy landscape of MoS_2 nanoflake on graphene. $Mo_{33}S_{66}$ nanoflake (similar in size to those in Fig 1) on graphene is computed to possess near-flat energy landscape with the highest difference between its maxima and minima as small as 84 meV. Such near-flat energy landscape is consistent with the MoS_2-graphene incommensurate contact.

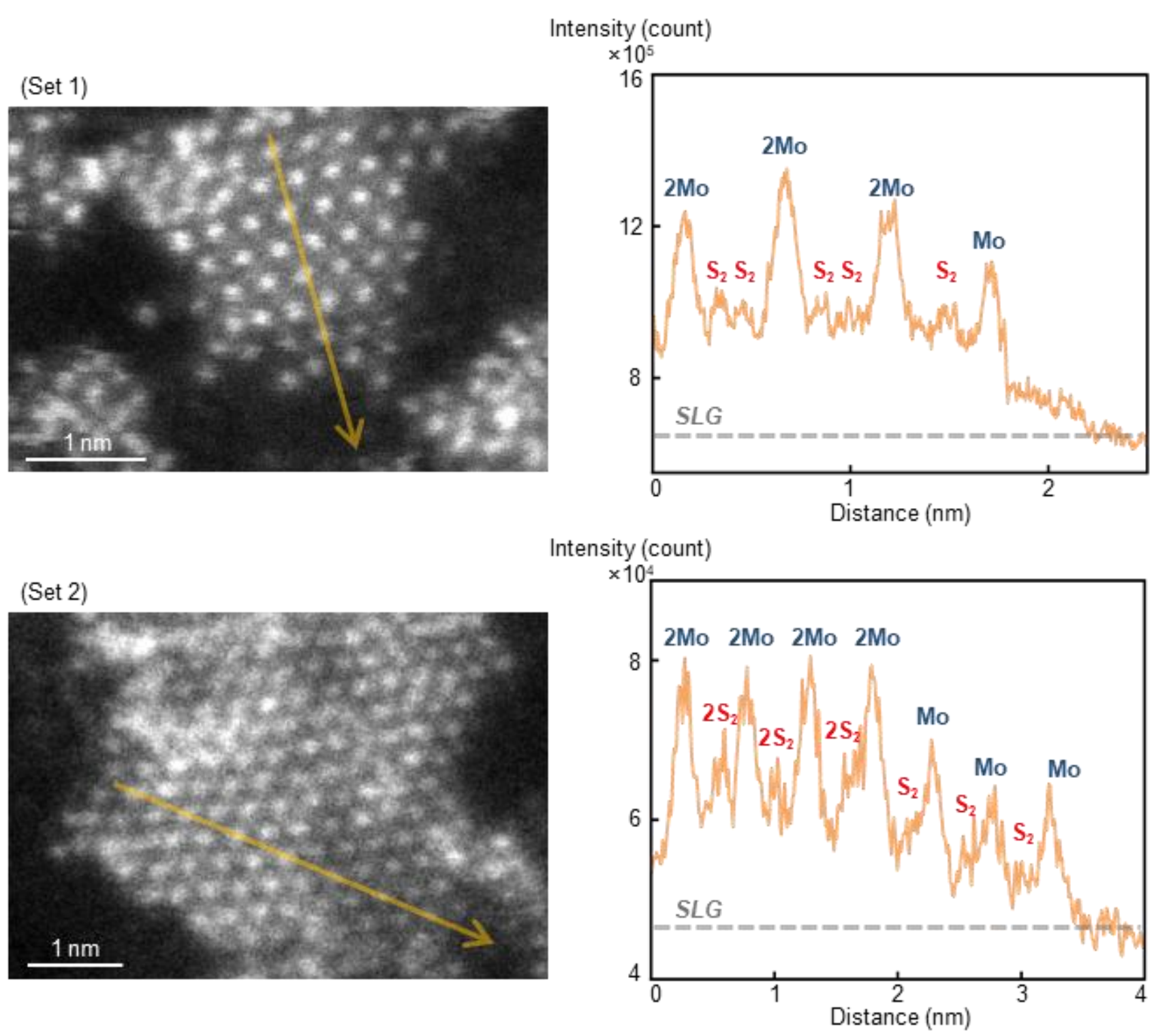

Fig. S5 | Initial states of the observed bilayer dynamics. Comparing STEM-HAADF image contrasts of S-atoms in the initial states shows that the Fig 2A case begins with an AB′-stacked bilayer MoS_2 while Fig 2B begins with an AA-stacked bilayer MoS_2. We confirm in each case by examining the STEM-HAADF contrast along the Mo-Mo axis (orange line), showing the presence of separate S_2-units between Mo-atoms in Fig 2A, confirming the AB′-stacking; while showing the presence of stacked S_2-units between Mo-atoms in Fig 2B, confirming the AA-stacking.

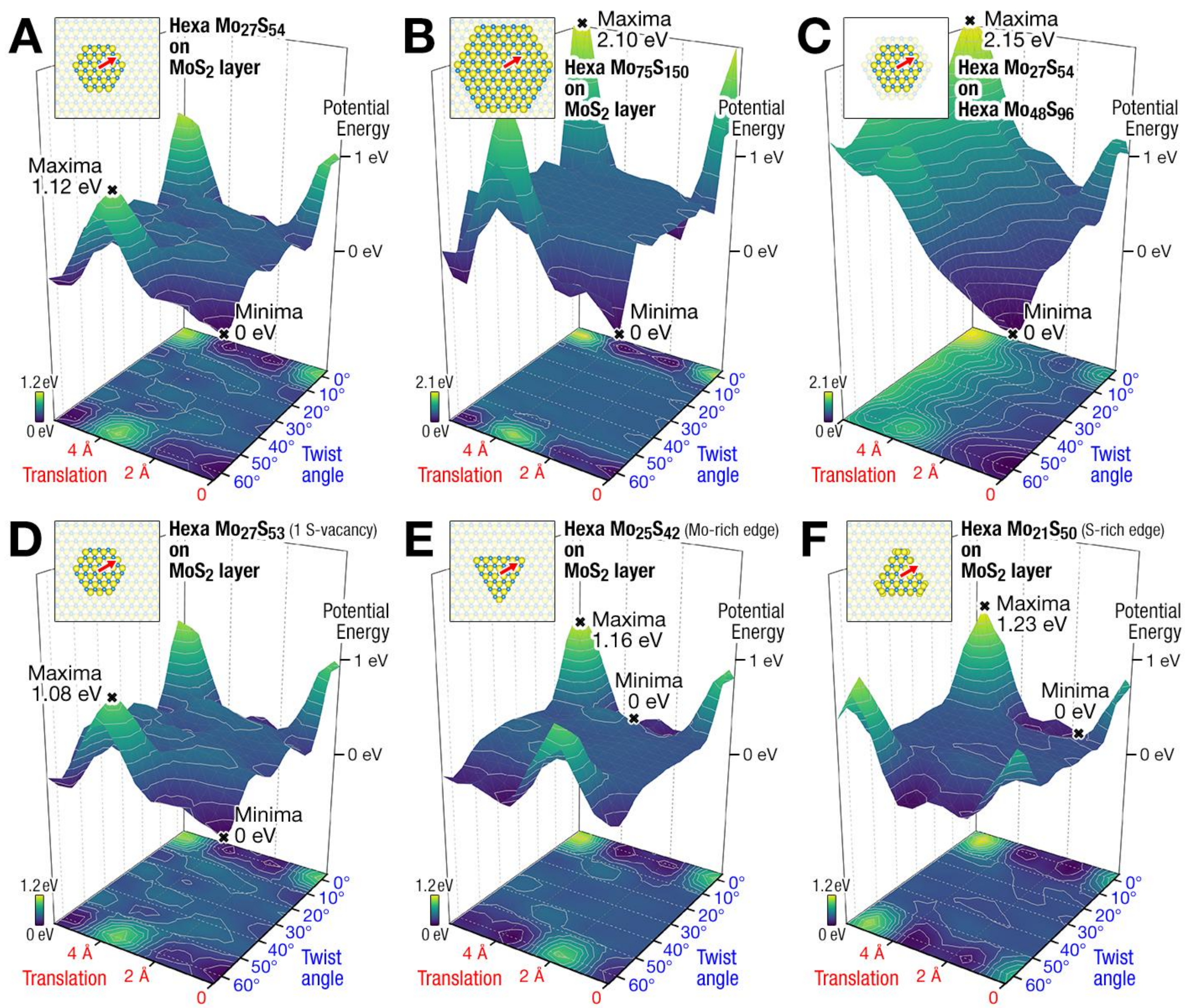

Fig. S6 | Computed potential energy landscape of various MoS_2 bilayer systems. The energy landscape is computed as a function of translation (0 to 6 Å) along the 'armchair' $[10\bar{1}0]$ direction (marked by the red arrow in every inset) and twist angle (θ_{twist}, 0° to 60°) for different upper-layer MoS_2 nanoflakes on a substrate: (A) Hexagonal-shaped $Mo_{27}S_{54}$ (similar in size to that in Fig 2A) on MoS_2 monolayer. (B) Larger hexagonal $Mo_{75}S_{150}$ on MoS_2 monolayer. (C) Hexagonal $Mo_{27}S_{54}$ on hexagonal $Mo_{48}S_{96}$. (D) Hexagonal $Mo_{27}S_{53}$ with a single S-vacancy defect on MoS_2 monolayer. (E) $Mo_{25}S_{42}$ with Mo-rich edges on MoS_2 monolayer. (F) $Mo_{21}S_{50}$ with S-rich edges on MoS_2 monolayer. The geography of the energy landscape is found to be similar in all cases, where the commensurate contact (θ_{twist} = 0° and 60°) gives a corrugated energy landscape along the translation coordinate, while the incommensurate contact ($\theta_{twist} \neq$ 0° and 60°) gives a near-

flat energy landscape along the translation coordinate. In the special case of flake-on-flake in (C), the energy landscape gives the same geography with an addition of increasing energy as the top flake is translated away from the bottom flake as a result of VdW attraction between the two nanoflakes. The flake-on-flake is noted to give potential wells consistent with experimental observation in Fig 3A, where θ_{twist} near 0° and 60° are energetic minima while θ_{twist} near 15° and 35° situated at near-flat landscape. In each case, energy values are relative to the minima (0 eV). The energies shown for (B) has been normalized by the nanoflake area (a factor of 0.36) to enable comparison with the smaller nanoflake.

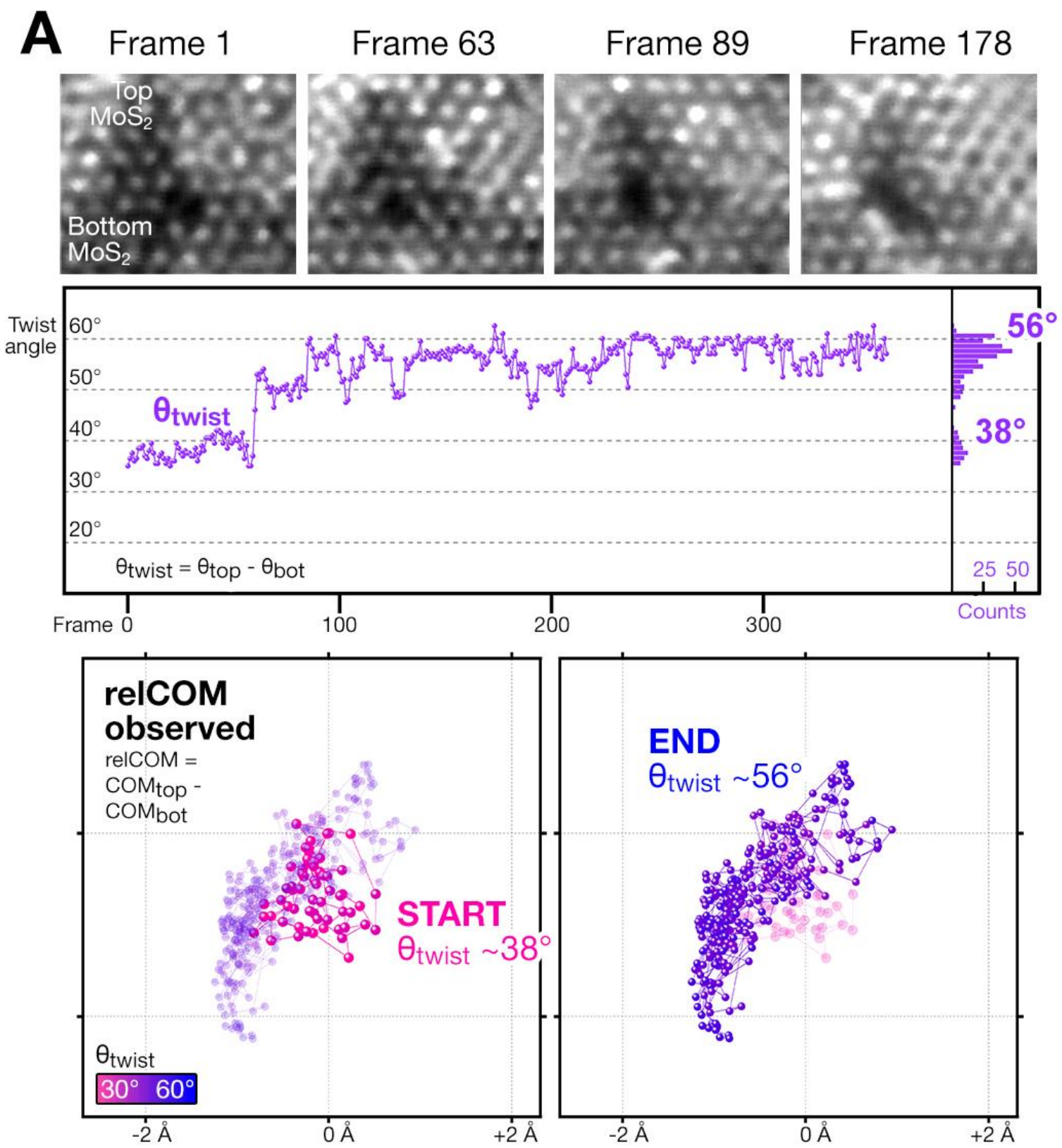

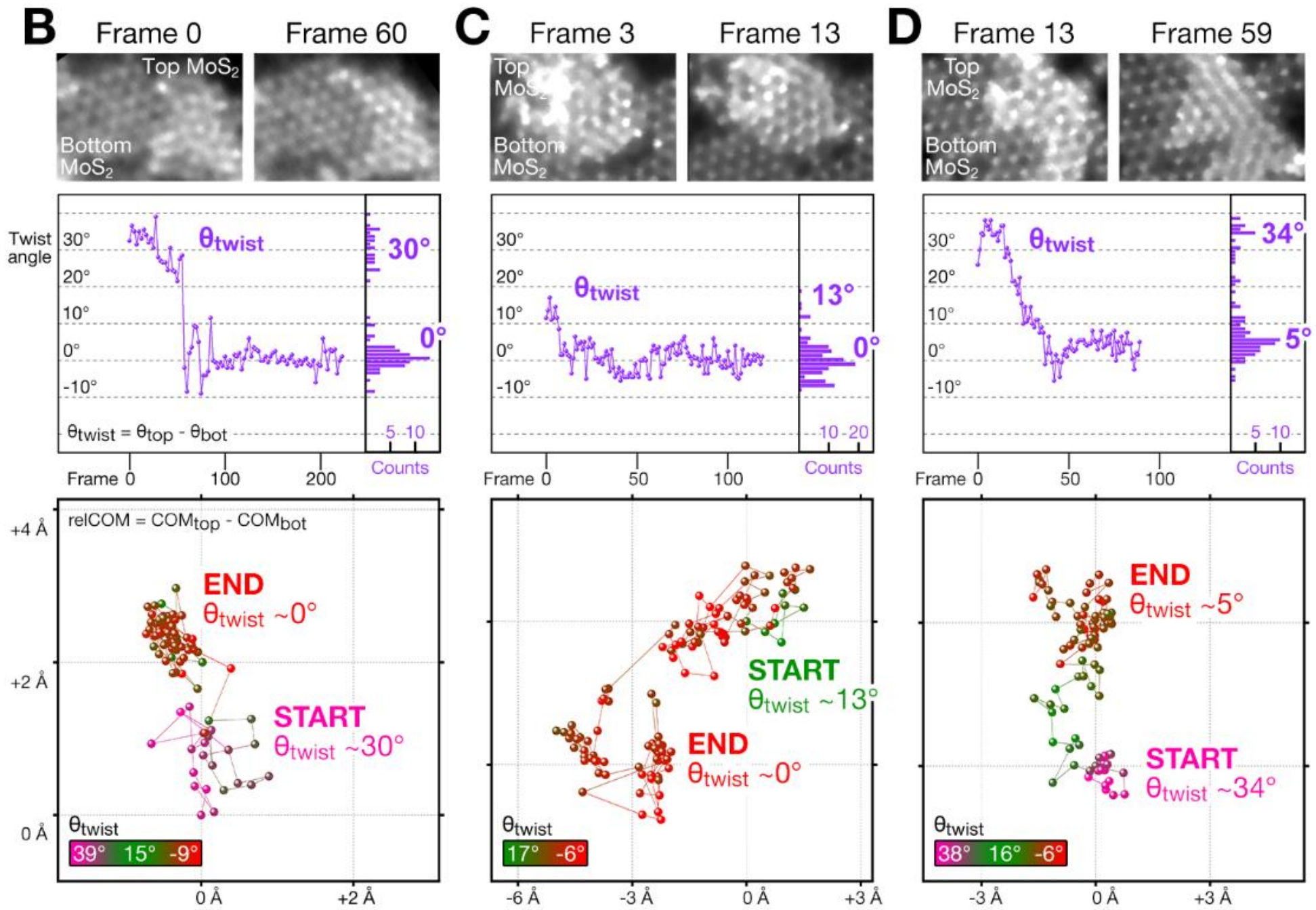

Fig. S7 | Additional cases of moiré superlattice dynamics observed. Similar to Fig 2, four cases of MoS_2 nanoflake were observed to rotate and translate above another MoS_2 monolayer with contact areas of 2.1 nm^2 for (A), 1.6 nm^2 for (B), 3.1 nm^2 for (C) and (D). Each case shows a transition from incommensurate moiré state into commensurate or near-commensurate states. Panel (A) shows the delocalized interlayer translation at both incommensurate θ_{twist} 38° and near-commensurate θ_{twist} 56°, whereby the latter is similar to that observed for near-commensurate θ_{twist} 4° shown in Fig 3B. Panel (B) shows transition from delocalized interlayer translation at θ_{twist} 30° to localized interlayer translation at θ_{twist} 0°. Panel (C) shows delocalized translation at near-commensurate θ_{twist} 5° and -5° before settling into localized translation at commensurate θ_{twist} 0°. Panel (D) shows delocalized translation at near-commensurate θ_{twist} 5° after transitioning from incommensurate θ_{twist} 34°.

Captions for Movies

Movie S1 | First observation case for single layer MoS_2 nanoflake on graphene. The recorded data was taken over 517 frames (~40 minutes) at a rate of 4.68 seconds per frame with acquisition breaks shown in Fig S2A. Scale: 100 pixels = 6.89 Å. The movie shows the frames at 15 frames per second.

Movie S2 | Second observation case for single layer MoS_2 nanoflake on graphene. The recorded data was taken over 549 frames (~42 minutes) at a rate of 4.68 seconds per frame with acquisition breaks shown in Fig S2B. Scale: 100 pixels = 6.63 Å. The movie shows the frames at 15 frames per second.

Movie S3 | First observation case for bilayer MoS_2 nanoflake on graphene. The recorded data was taken over 530 frames (~41 minutes) at a rate of 4.68 seconds per frame with acquisition breaks shown in Fig S2C. Scale: 100 pixels = 6.89 Å. The movie shows the frames at 15 frames per second.

Movie S4 | Second observation case for bilayer MoS_2 nanoflake on graphene. The recorded data was taken over 464 frames (~36 minutes) at a rate of 4.68 seconds per frame with acquisition breaks shown in Fig S2D. Scale: 100 pixels = 10.56 Å. The movie shows the frames at 15 frames per second.
